# How usefulness shapes neural representations during goal-directed behavior

**DOI:** 10.1126/sciadv.abd5363

**Published:** 2021-04-07

**Authors:** G. Castegnetti, M. Zurita, B. De Martino

**Affiliations:** 1 Institute of Cognitive Neuroscience, University College London, London, UK.; 2 Wellcome Centre for Human Neuroimaging, University College London, London, UK.

## Abstract

Value is often associated with reward, emphasizing its hedonic aspects. However, when circumstances change, value must also change (a compass outvalues gold, if you are lost). How are value representations in the brain reshaped under different behavioral goals? To answer this question, we devised a new task that decouples usefulness from its hedonic attributes, allowing us to study flexible goal-dependent mapping. Here, we show that, unlike sensory cortices, regions in the prefrontal cortex (PFC)—usually associated with value computation—remap their representation of perceptually identical items according to how useful the item has been to achieve a specific goal. Furthermore, we identify a coding scheme in the PFC that represents value regardless of the goal, thus supporting generalization across contexts. Our work questions the dominant view that equates value with reward, showing how a change in goals triggers a reorganization of the neural representation of value, enabling flexible behavior.

## Teaser

The activity of the human prefrontal cortex automatically encodes the usefulness of an item according to the current goal.

## INTRODUCTION

Imagine being stranded on a deserted island after crashing a cargo aircraft. You need to choose between a metal chair and a wooden chair to light a fire to keep yourself warm. This goal is very different from that which you would normally have when choosing between chairs (i.e., sitting down comfortably or with style). The wooden chair would be very good for burning but might be uncomfortable, and the metal chair would be useless for burning and might not be stylish. They share the conceptual space of “furniture for sitting,” but they widely differ in other conceptual subspaces (e.g., material and style).

Despite all these differences, our brains can readily provide an answer to these conundrums—presumably by mapping the options’ value into a common currency under which a meaningful comparison can take place (*1*, *2*). In the past two decades, our understanding of the neural circuitry underpinning these value-based decisions has greatly increased. This established a primary role for the ventromedial prefrontal cortex [vmPFC; (*1*, *3*–*5*)], although the algorithmic implementation of this value computation has, so far, remained elusive. Furthermore, in most of these previous studies, the goal was not directly manipulated, and the stimuli were always evaluated in terms of monetary value (*1*, *6*, *7*) or subjective pleasantness (*1*, *8*–*10*). Notably, several studies have investigated context-dependent value, focusing on how contexts affect value construction and deployment during evaluation (*11*, *12*), choice (*13*–*15*), or learning (*16*). However, in most of these studies, the context manipulation was clearly defined and required an explicit evaluation or revaluation of the options. Other studies have shown that change in state (e.g., satiety and mood induction) provides a powerful contextual manipulation that affects the construction of value behaviorally (*17*) and neurally (*18*, *19*) but was always associated with the hedonic evaluation of the options. Nonetheless, in most real-life situations, the value of an action is strongly tied to the behavioral goal that an agent seeks to achieve (*20*). This should be independent from the hedonic nature of value, and it has to rapidly change when the goal changes. In this study, we test whether changing a goal triggers a reorganization of perceptual information even in the absence of an explicit evaluation or choice. Furthermore, we show that this remapping happens on a rapid time scale (i.e., the goal switches repeatedly during the task), under a top-down control (i.e., participants actively switch goals), and that humans can perform this flexibly for very abstract scenarios never encountered before (e.g., imagine using a pair of shoes to light a fire). To do so, we devised an experiment in which human volunteers underwent functional neuroimaging while imagining using different items to achieve two distinct goals. In short, participants were asked to picture themselves as pilots of a cargo aircraft flying over the ocean at night. A sudden engine failure required an emergency landing on a deserted island. The final objective of the task was to flee the island: To do so, participants could use each of the items retrieved from the aircraft wreck. We proposed two possible escape strategies, each requiring the achievement of a separate goal: either starting a fire to be detected by a rescue team (hereafter referred to as burning goal) or keeping a boat anchored ashore until morning (anchoring goal).

To test the effect of goal manipulations on brain activity, we analyzed functional magnetic resonance imaging (fMRI) data with a univariate approach and with representational similarity analysis (RSA), a multivariate technique suited for the study of the representational content of brain activity patterns (*21*, *22*). To build these representations, participants had to consider the consequences of their actions rather than applying fixed stimulus-action maps, the hallmark of model-based computations (*23*). This required perceptually identical stimuli to flexibly rearrange into new, goal-dependent representations that emphasize goal-relevant features (*24*). Since value-based decisions build on internal estimations of goal-dependent value (termed “usefulness” here), as well as confidence in these estimations (*10*, *25*–*27*), we tested the hypothesis that item valuations are supported by brain representations that organize elements according to these abstract attributes. We then sought to extend the common currency hypothesis by proposing that such currency is common not only across item categories (*1*, *2*, *4*) but also across goals. In line with a previous approach (*28*), we tested this with a classification algorithm, showing that the vmPFC encodes a distributed code for usefulness that is independent of the decision-maker’s current goal.

## RESULTS

### Behavioral results

**Fig. 1:**
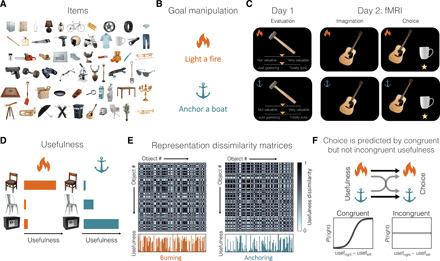
Task and behavioral measures. (**A**) Sample (7 of 120) of the items presented in the task. (**B**) The task involved two separate goals: lighting a fire (burning goal: orange flame) and anchoring a boat (anchoring goal: teal anchor). In the remainder of the paper, these goals are associated with the presented icons and colors. (**C**) On day 1, subjects evaluated each item’s usefulness for each goal and reported their confidence in the evaluation (evaluation trials). On day 2, we acquired fMRI data while participants experienced the items of day 1 either in isolation (imagination trials) or in pairs (choice trials). During imagination trials, they were tasked to imagine using the item for the current goal. During choice trials, they were tasked to choose the item they deemed more valuable for the current goal; a yellow star under the chosen item confirmed the selection. (**D**) Subjective valuations depended on the decision-maker’s goal, as exemplified here. A wooden chair is useful for burning, unlike a metal chair (although perceptually similar) or a safe box. In contrast, the safe box and, to some extent, the metal chair, might be useful for anchoring, unlike the wooden chair. (**E**) Dissimilarity matrices between items under the two goals, built on estimations of a sample participant. Each entry of the matrices indicates the absolute difference in subjective usefulness (plotted below the matrices in orange and teal) between a pair of items. (**F**) Top: We separately tested the relation between choice and the usefulness reported by participants for the same goal (congruent case; black arrow) or the alternative goal (incongruent case; gray arrow). Bottom: On day 2, choices were predicted by the usefulness assigned on day 1 for the congruent goal but were unrelated to the incongruent goal (graphs for illustration; subjects’ performances are presented in fig. S1).

On day 1, we acquired subjective valuations of a set of 120 items (Fig. 1A) in three different sessions. During the first session, participants (*n* = 30) indicated how familiar they were with each item and estimated its monetary value. During the second and third sessions, participants evaluated the usefulness of each item to achieve two goals—starting a fire (burning goal) and anchoring a boat (anchoring goal)—and reported their confidence in such valuation (Fig. 1, B to D). Shared correlations between these estimations are listed in Table 1. The usefulness and confidence assigned to the same items in the context of different goals shared little variance [value: 1.44%, correlation coefficient (*r*) = 0.12; confidence: 0.16%, *r* = 0.04; see Fig. 1E for usefulness and confidence and the ensuing dissimilarity matrices from a sample subject]. Moreover, we found a quadratic relationship (β = 0.03, *P* < 0.001) between goal-dependent confidence and usefulness in line with previous reports of confidence in value-based decision-making (*10*, *26*). The variance between monetary value and usefulness for burning (0.36%, *r* = −0.06) or anchoring (2.89%, *r* = 0.17) was also small. Obtaining a weak correlation between these scores was critical to clearly differentiate their signature effect on behavior and brain activity.

**Table 1:** Correlation between behavioral measures. Correlation coefficients (Pearson’s *r*) between congruent and incongruent usefulness and confidence and monetary value. Data are presented as means ± SD and the range [minimum, maximum].

***U*_anchoring_**	***C*_burning_**	***C*_anchoring_**	***V*_monetary_**	
0.12 ± 0.22 [−0.23, 0.54]	0.11 ± 0.21 [−0.17, 0.58]	−0.02 ± 0.14 [−0.40, 0.23]	−0.06 ± 0.09 [−0.21, 0.16]	*U*_burning_
	0.04 ± 0.13 [−0.30, 0.31]	−0.22 ± 0.26 [−0.67, 0.20]	0.17 ± 0.12 [−0.08, 0.40]	*U*_anchoring_
		0.04 ± 0.11 [−0.13, 0.29]	0.02 ± 0.08 [−0.17, 0.21]	*C*_burning_
			−0.03 ± 0.11 [−0.33, 0.09]	*C*_anchoring_

On day 2, participants underwent fMRI scanning while engaging with two kinds of trials: imagination and choice trials (Fig. 1C). In imagination trials (71.4% of the trials), they were asked to vividly imagine how to use an item to achieve the proposed goal. Since we could not control the degree of imagination vividness that participants experienced in the task, they were specifically instructed to imagine using each item as vividly as possible, even if objects had a very low usefulness (see the instructions delivered to the participants in the Supplementary Materials). In choice trials (28.6% of the trials), they had to indicate which of two proposed items best served the current goal. The average reaction time for choice was 1.47 s, and it was not affected by goal manipulation (*t*_29_ = −0.41, *P* = 0.69), demonstrating that the two goals were approximately balanced in terms of difficulty. A logistic regression at the group level revealed that choices were predicted by the difference between usefulness (Δ*U* = *U*_R_ − *U*_L_) assigned on day 1 for the congruent goal (β = 4.71, *P* < 0.001; Fig. 1F), even when considering participants excluded from the rest of the analyses because of their inconsistent choices (β = 4.02, *P* < 0.001; see Methods). Choices were not predicted by the usefulness assigned for the incongruent goal (β = −0.11, *P* = 0.34; Fig. 1F) nor by monetary value (β = −4.0 × 10^−8^, *P* = 0.99). Participants’ choices were also affected by the difference in confidence between the items (Δ*C* = *C*_R_ − *C*_L_, β = −0.64, *P* = 0.001). No significant interaction was found between Δ*U* and Δ*C* (β = 0.69, *P* = 0.22).

### Neuroimaging: Univariate analyses

**Fig. 2:**
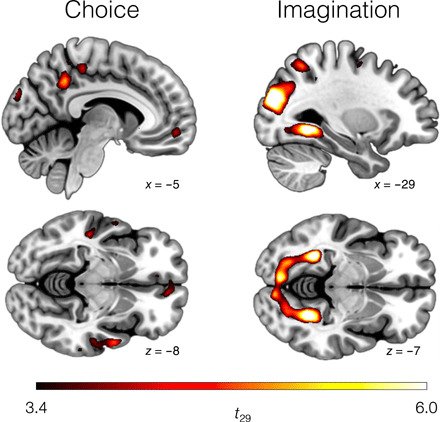
Results of the univariate analysis. For choice, the displayed clusters indicate activity that positively correlated with the signed difference between the value of the chosen and unchosen item. For imagination, the clusters indicate activity positively correlated with the congruent value of the presented item. All clusters were identified with an uncorrected threshold of *P* < 0.001 and then FWE-corrected at *P* < 0.05.

Behavioral data demonstrated that participants’ choices were guided by the items’ usefulness toward the current goal (i.e., congruent usefulness) but not the value assigned under alternative goals (i.e., incongruent usefulness and monetary value; Fig. 1F). We therefore hypothesized that activity in the vmPFC—a brain region routinely associated with value computation and choice (*26*, *29*, *30*)—would be also modulated by the congruent usefulness only. To test this hypothesis, we regressed the blood oxygen level–dependent (BOLD) signal observed during choice (Fig. 1C) against the signed difference between chosen and unchosen usefulness (Δ*U*), computed using estimates for congruent and incongruent goals. We also regressed the choice BOLD signal against the signed difference between the monetary value subjectively estimated for the chosen and unchosen value. All the reported activation clusters were identified with an uncorrected threshold of *P* < 0.001 and corrected for family-wise error (FWE) at the cluster level at *P* < 0.05. We found that vmPFC activity positively correlated with congruent Δ*U* {peak voxel in Montreal Neurologic Institute (MNI) space: [−8, 54, −8], *t*_29_ = 4.91, *P* < 0.001; Fig. 2} but was unrelated to incongruent usefulness or monetary value, even at a liberal threshold (*P* < 0.01 uncorrected; fig. S2). We found no signal proportional to incongruent or monetary value even after restricting the search to a region of interest (ROI) defined within a 10-mm radius around the peak of the vmPFC activation related to congruent usefulness or around the vmPFC peak activation from a previous independent study {[−3, 47, −8]; (*30*)}. These results suggest that the value signal in the vmPFC activity is not an absolute property of the item related to subjective preferences but is determined by the item’s usefulness toward the decision-maker’s current goal.

Next, we analyzed imagination trials (Fig. 1C). We found a parametric response to the item’s usefulness in a large cluster including the lingual and fusiform gyrus (FWE-corrected at the cluster level: [32, −44, −8], *t*_29_ = 7.85, *P* < 0.001; Fig. 2) and bilaterally in the middle occipital gyrus extending into the superior occipital gyrus (left: [−30, −86, 24], *t*_29_ = 7.65, *P* < 0.001; right: [32, −78, 24], *t*_29_ = 6.33, *P* < 0.001). Intriguingly, the activity in these areas correlated also with incongruent usefulness (right: [30, −50, −6], *t*_29_ = 8.24, *P* < 0.001; left: [32, −82, −18], *t*_29_ = 7.84, *P* < 0.001; fig. S2) and monetary value (right: [16, −90, 2], *t*_29_ = 5.78, *P* < 0.001; left: [−24, −82, −8], *t*_29_ = 5.27, *P* < 0.001; fig. S2), even if these three definitions of value were largely uncorrelated (Table 1). Since participants were aware of all of the all goals involved in the task (due to valuations on day 1), this result is consistent with a value-driven attentional capture by goal-irrelevant values (*30*). No activity was found in the vmPFC or posterior cingulate cortex nor in other prefrontal areas, even at liberal thresholds (*P* < 0.01 uncorrected), suggesting that the involvement of these regions is limited to situations in which usefulness triggers a behavioral response (e.g., choice or explicit evaluation).

### Neuroimaging: Multivariate analyses (RSAs)

From behavior and neuroimaging data, we showed that both choice and brain activity are modulated by the decision-maker’s goal. We then sought to investigate in more detail this flexible mapping of stimuli into behavior. We predicted that visual areas support goal-independent item representations that are determined by the item’s perceptual features but are insensitive to behavioral goal. On the contrary, we hypothesized that higher-level association areas, such as the vmPFC, would represent items in a novel abstract space that integrates item and goal, such as the degree of usefulness of the item and the associated confidence level. We tested these hypotheses with RSA (*21*, *22*). This is a multivariate analysis approach suited for testing the representational content of brain activity patterns. In the RSA, we first compute the dissimilarity between the multivoxel brain activity elicited by the different stimuli. The metric used to evaluate the dissimilarity between stimuli is *d* = 1 − Pearson’s *r* (*21*, *22*). These dissimilarities are then organized in a representational dissimilarity matrix (RDM), which is then tested against model RDMs that encode the representational distances expected under different candidate metrics (e.g., difference in usefulness).

### Goal-independent representations

**Fig. 3:**
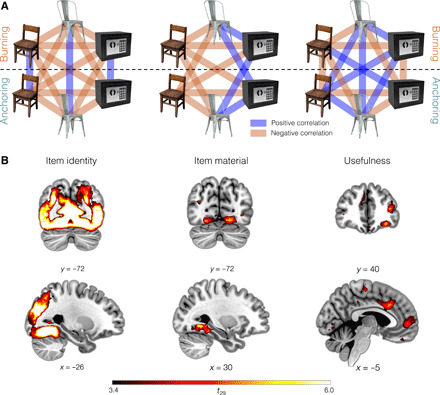
Results from the RSA searchlights. (**A**) Schematic representation of the representations of item identity, material, and usefulness. Blue and red lines indicate positive and negative correlations, respectively. In the central panel, the lines connecting the same item across goals are absent as the corresponding elements were removed from the RDM to ensure independence between item material and identity (see Methods). (**B**) Brain regions whose activity follows the above representations, as identified with a volumetric searchlight. All the displayed clusters were detected with a cluster definition threshold of *P* < 0.001 and FWE-corrected with a permutation test at *P* < 0.05. Brain image templates, copyright 137 (C) 1993–2004 Louis Collins, McConnell Brain Imaging Centre, Montreal Neurological Institute, McGill University.

We reasoned that a purely perceptual representation (including high-level object identity) would be unaffected by the goal manipulation and thus exhibit high similarity across presentations of the same item during imagination trials regardless of the context in which such presentation occurred and low similarity between different items (i.e., *d_ij_* = 1 − δ*_ij_*, where δ is the Kronecker delta and *i* and *j* are item labels; Fig. 3A). To topologically localize these representations, we used a volumetric searchlight in which the correlation between model- and brain-based RDMs was computed within a 9-mm-radius spherical region centered in each voxel of the subjective gray matter mask. The obtained correlation coefficient (Spearman’s ⍴) was then assigned to the central voxel, yielding subjective correlation maps on which we lastly computed group-level statistics with a nonparametric permutation test at the cluster level: All the reported clusters were identified with a cluster detection criterion of *P* < 0.001 and FWE-corrected at *P* < 0.05 (*31*). Activity consistent with a perceptual representation was found in a single large cluster (peak voxel in MNI space: [28, −54, −12]; *t*_29_ = 16.1, *P* < 0.001; Fig. 3B) encompassing portions of the occipital lobe and extending into the temporal lobe, including primary visual areas and higher-level structures of the ventral stream like the fusiform and the inferotemporal gyrus, known to be involved in the conceptual processing of pictures of real objects (*32*).

Next, we reasoned that for both the burning and the anchoring goal, an intermediate step of this valuation process could be the assessment of material composition. We thus asked an independent set of participants to evaluate the relative content of wood, metal, plastic, and fabric of all the items. Distances in this four-dimensional compositional space were used to define a new model RDM, which reflected the pairwise difference in material composition between the two items (Fig. 3A). We found material composition representation during imagination trials in the inferior temporal gyrus (ITG; [8, −78, −8]; *t*_29_ = 8.43, *P* < 0.001; Fig. 3B), in agreement with previous studies reporting that material textures could be decoded in this brain area (*33*). Our results add to these findings by showing that material composition can be extracted by pictures of real-life items (instead of pure textures) and that representational similarity is proportional to the continuous difference in composition and not just a binary representation of whether items belong to the same material category.

### Goal-dependent representations

We then performed a series of multivariate analyses to specifically test the hypothesis that goal-dependent valuation (i.e., usefulness) is supported by the construction of flexible representations, where items that are similarly useful are represented similarly. For example, if the decision-maker’s goal is lighting a fire, then a metal- and a wood-made chair, while perceptually similar, are dissimilar in the usefulness space (Fig. 1D). In the brain, this would entail a representation in which distances between activity patterns scale with the difference in usefulness. In particular, we hypothesize that the vmPFC—because of its role in value computation (*34*, *35*) and in the deployment of goal-relevant associative networks (often termed schemas) (*36*)—supports these goal-dependent deformations of the neural representations. We investigated this with a searchlight procedure on data collected during imagination trials, aimed at detecting brain representations encoding the absolute differences in subjective rating of usefulness collected on day 1 (i.e., *d_ij_* = ∣*DV*∣*_ij_* = ∣*V_i_* − *V_j_*∣, with *V_i_* and *V_j_* value of items *i* and *j*, respectively). We found representations meeting this criterion in a number of prefrontal subregions, including the vmPFC (peak voxel in MNI space: [−10, 54, 2]; *t*_29_ = 5.04, *P* = 0.036) and the orbitofrontal cortex (OFC; [−32, 38, −14], *t*_29_ = 5.75, *P* = 0.004; Fig. 3B). The OFC is another central node of the valuation circuit (*34*, *35*) and is implicated in inferring value on the fly from mental simulation (*37*, *38*). We detected a similar representation also in the dorsolateral PFC (dlPFC) extending into the left insula ([−40, 50, 12], *t*_29_ = 5.69, *P* = 0.006), previously implicated in context-dependent valuation (*13*). Last, representations of usefulness were also found in the lingual and fusiform gyrus ([−24, −48, −6], *t*_29_ = 7.51, *P* < 0.001) extending into the parahippocampal gyrus and the hippocampus and in the inferotemporal cortex ([52, −52, −16], *t*_29_ = 7.51, *P* < 0.001). We found no evidence of brain representations correlating with the representations of incongruent usefulness or monetary value. Crucially, these analyses were restricted to imagination trials, during which participants produced no overt responses, suggesting that goal-relevant representations emerge even in the absence of an explicit behavioral output. Moreover, we found no univariate signal in prefrontal areas during imagination trials (Fig. 2), suggesting that the activation pattern in these regions changes in shape—as a function of the usefulness of the imagined item—without substantially altering the overall average activation level.

### The OFC displays an integrative code of value and confidence

**Fig. 4:**
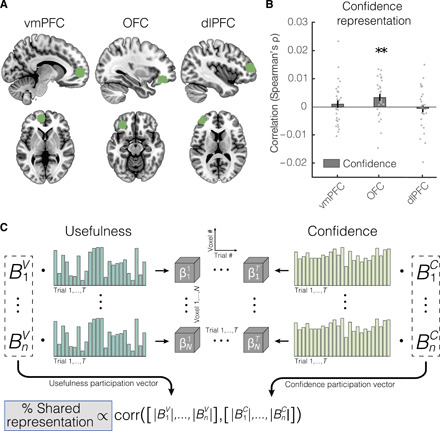
Confidence analyses. (**A**) ROIs used for confidence analyses. These ROIs were defined in correspondence with the statistically significant clusters obtained from the correlation map for usefulness (vmPFC, OFC, and dlPFC) drawn by the searchlight. (**B**) Correlation between the confidence representation and the brain activity in the three prefrontal areas that represented usefulness. **P < 0.01. (**C**) Analysis pipeline for the ROI-based analysis to quantify the overlap between the neural schemes of usefulness and confidence. The matrix in the center of the figure represents the activity vector (vertical dimension) of the *N* voxels in the ROI over *T* trials (horizontal dimension). The trial-by-trial activity of each voxel (i.e., each row of the matrix, which has length *T*) was then linearly regressed against the vectors of subjective estimates of usefulness and confidence in each trial. Each of these regressions resulted in a coefficient for each voxel; since we did two regressions per voxel—one for usefulness and one for confidence—this resulted in two coefficients for each voxel, describing the linear relation between the voxel activity with usefulness and confidence (BiU and BiC, respectively, with *i* = 1,…,*N*). The absolute values of these coefficients were interpreted as the degree to which the corresponding voxel participates in the representation of usefulness and confidence and were grouped in two participation vectors, which summarized the extent to which the voxels in the ROI took part in the representation of usefulness and confidence. The correlation between the two participation vectors was then taken as an indication of whether the representations of usefulness and confidence were supported by the same neural code or by overlapping but distinct codes (*42*, *43*).

Our behavioral data show that confidence on the usefulness estimations also affects behavior, adding to mounting evidence showing that confidence is intimately linked with value in guiding decisions in both animals (*39*) and humans (*10*, *25*, *26*). This implies that, at some step of the transformation of input stimuli into behavior, usefulness and confidence are integrated. Therefore, we sought evidence of neural activity underpinning this integrative code. To this end, we conducted an RSA within the prefrontal areas that displayed usefulness representations in the vmPFC, OFC, and dlPFC (Fig. 4A), as resulted from the searchlight analysis (see previous section). This analysis was restricted to these brain regions because a representation of a common value currency has been reported predominantly in prefrontal regions (*1*, *40*). However, we believe that goal-dependent abstractions are likely to be supported by a distributed network, and therefore, they might not be localized exclusively in these regions but extend to other brain areas that do not display goal-dependent usefulness representation and are therefore not considered here. See table S2 for more details about the areas that showed goal-dependent value representations.

A significant correlation between confidence and neural activity was found in the OFC (one-sided Wilcoxon signed-rank test, *P* = 0.003; Fig. 4B). On the contrary, both the vmPFC (*P* = 0.36) and the dlPFC (*P* = 0.61) did not show a significant correlation between BOLD signal and confidence (Fig. 4B). However, since we did not detect a significant region main effect [one-way analysis of variance (ANOVA), *P* = 0.109], the difference among these regions needs to be interpreted with caution. Here, we only tested for confidence representation and thus make no statistical claims about usefulness, meaning that the inference is not circular. Furthermore, the subjective estimates of usefulness and confidence shared only 0.49% of the variance (*r* = −0.07 across goals; within-goal correlations are presented in Table 1), suggesting that this code is unlikely to reflect the behavioral correlation between the two scores.

It has been suggested that, during decision-making, confidence and the related decision accuracy are inherently valuable and might be automatically integrated in the value signal (*10*). Therefore, we then asked whether these representations were supported by the same neural population or by distinct populations, which would provide an indication of whether these quantities are integrated in the OFC or are encoded as separate independent variables by two distinct populations in the OFC. To do so, we adapted an analytic approach previously used to analyze multiunit firing patterns in rodents (Fig. 4C) (*41*, *42*). Specifically, we computed “voxel participation vectors,” which indicated the extent to which each voxel in an ROI is involved in representing usefulness or confidence; a positive correlation between the two vectors would be suggestive of a shared neural code. Since the distribution underlying this correlation was unknown, we drew our statistical inference using a permutation test by repeating the analysis 10,000 times after randomly permuting the voxel labels, in line with previous approaches (*41*–*43*). We found that voxel participation vectors from usefulness and confidence in the OFC were positively correlated (ρ = 0.053; two-sided permutation test, *P* = 0.004). When applied to vectors of voxels instead of neural populations, this analysis is not conclusive, as a positive correlation might still reflect the activity of distinct populations within the same voxels. However, it provides some evidence in support of the hypothesis that neural populations that support the representations of value and confidence in the OFC might at least partly coincide (*10*) and that is consistent with the theoretical proposal that confidence naturally emerges as a balance of evidence during a comparison process itself (*26*).

### The vmPFC encodes a common currency for usefulness

Our results show that the vmPFC represents items according to their goal-dependent usefulness. In the vmPFC, values have been suggested to be represented on a common scale (or “currency”) that allows different item values to be mapped on a common scale and compared. This was suggested in most previous studies by demonstrating that the vmPFC showed a similar activation as a function of value even across different categories of items [e.g., food or nonconsumable items; (*1*, *4*)]. This appears to be true not only when looking at the average vmPFC activity but also for the multivoxel activation pattern (*28*). Crucially, however, humans are also able to compare usefulness across different goals. For example, we are able to determine whether a wooden chair would be more useful for burning than a metal chair for anchoring. To do so, the brain must first compute the usefulness of the two chairs—the first chair for burning and the second for anchoring, respectively—and then bring this usefulness to a common scale for comparison. This requires the vmPFC to encode a distributed usefulness code that is independent of item category and goal. For example, an item with usefulness *X* for goal A would be represented similarly to another item with usefulness *X* for goal B. Given our operational definition of usefulness in terms of goal-dependent value, we thus expected that if the vmPFC is critical to support a common currency for subjective preferences (*1*, *28*, *29*, *44*), then it might also support a representation of usefulness that is independent of the current goal (i.e., whether the goal is burning or anchoring). To test this hypothesis, we used a cross-classification procedure.

**Fig. 5:**
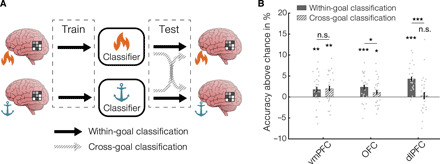
Cross-classification. (**A**) Schematics of the cross-classification procedure. Goal-specific classifiers were trained to classify multivoxel brain activity into either high- or low-value categories. Next, we computed the accuracy of these classifiers in classifying the brain data acquired either during the same goal (within-goal classification) or the alternative goal (cross-goal classification). (**B**) Average classifier accuracy in categorizing multivoxel brain activity into high- or low-value trials, for activity acquired under the same goal or the alternative goal. *P < 0.05; **P < 0.01; ***P < 0.001. n.s., not significant.

Specifically, for each of the two goals, we trained pattern classifiers to categorize the multivoxel activity into low- or high-usefulness items and then computed the classification accuracy on data either from the same goal (within-goal classification) or from the alternative goal (cross-goal classification; Fig. 5A). Similar within- and cross-classification accuracies would indicate that, although items’ usefulness changes with goal as we saw above, the neural code supporting its representation is conserved across goals. As in the previous section, we performed this analysis in the prefrontal ROIs where usefulness representation was strongest, namely the vmPFC, OFC, and dlPFC (Fig. 4A). For each participant, we built six classifiers, one for each goal and ROI. Not unexpectedly, since the analysis was performed in correspondence of the usefulness representations detected with the RSA, within-goal classification accuracy was above chance in all three ROIs (vmPFC: 51.8%, one-sided Wilcoxon signed-rank test, *P* < 0.01; OFC: 52.4%, *P* < 0.001; dlPFC: 54.3%, *P* < 0.001; Fig. 5B). In contrast, cross-classification accuracy was above chance in the vmPFC (52.0%, *P* < 0.01) and OFC (51.1%, *P* < 0.05) but not in the dlPFC (50.0%, *P* = 0.35; Fig. 5B).

While cross-classification in the OFC was above chance but still lower than within-goal accuracy (*P* < 0.05), cross-classification in the vmPFC was indistinguishable from within-goal accuracy (*P* = 0.55; Fig. 4). Note, however, that the ANOVA analysis over the results showed that there is no significant region by cross/within interaction (*P* = 0.386). Therefore, further analyses would be needed to assess whether there is a difference between these areas. This would be consistent with a previous study reporting that the vmPFC, but not the OFC, uses a common scheme to represent item from different categories (*28*) and suggests that the neural code supporting usefulness representation in the vmPFC is maintained across goals and might be critical to enable comparison and choice between incommensurable behavioral options. Note that from the searchlight analysis, we found that representations in the vmPFC are more similar for items with similar usefulness. This can be because of similarity within goals: Items with similar usefulness for burning are represented similarly, but their representation might differ from items that have comparable usefulness for anchoring. With this analysis, instead, we explicitly addressed the similarity across goals, testing to what extent items with similar usefulness across goals are represented similarly. In summary, while usefulness is clearly goal dependent, here we found evidence that the representation of, for instance, “high usefulness,” is conserved across goals in the vmPFC, while we found no evidence of this being the case in the OFC and dlPFC. While the cross-classification performance in the vmPFC suggests the presence of universal usefulness coding, the negative results in the OFC and dlPFC could also be affected by the precise definition of the ROIs, which might include goal-dependent components lowering the cross-correlation performance without ruling out some universal coding within the ROI. This analysis was restricted to the prefrontal ROIs where we found usefulness encoding from the RSA analysis and therefore does not exclude the presence of universal encoding elsewhere in the brain.

## DISCUSSION

Here, we studied how the human brain flexibly adapts its neural representation of usefulness—defined as goal-dependent value—of everyday-life items in response to changes in the decision-maker’s goal. From behavioral data, we showed that participants’ choices were exclusively predicted by the subjective value that they assigned to the options in the context of a congruent goal. With neuroimaging, we found that activity in the vmPFC correlated with the degree of usefulness to achieve the current goal but was unrelated to the value for alternative goals. Previous work has shown that activity in the vmPFC tracks the value of the available options or actions (*1*, *4*, *10*, *26*, *30*). However, in most studies, value was defined in monetary terms (e.g., willingness to pay) or in terms of subjective pleasantness (e.g., liking rate), overlooking the fact that the value of an action is critically dependent on the decision-maker’s goal. This left unanswered the question of whether value signals in the brain reflect expected reward or an operational construct such as goal-directed usefulness. If value does indeed depend on goal, then the mapping of sensorial inputs into behavioral outputs must be flexible. Since nature does not provide explicit labels, the criteria used to build value in a given context can become obsolete as soon as the context changes. This implies that a change of context generally entails a change in the goal-dependent value. If your car speeds toward a cliff, then breaking your bones to jump out alive becomes a desirable option, while no amount of money would be of any use. We thus propose the following interpretation of value and its associated neural computations: The value of an action is defined by the extent to which it fulfills the current goal. To this end, the representations of the available options must emphasize the features that are relevant for the goal and de-emphasize the others. To Abraham Maslow’s famous quote, “If all you have is a hammer, everything looks like a nail,” we might thus add that if your goal is hammering a nail, everything looks like a hammer.

To test these hypotheses, our design decouples value from its hedonic or rewarding attributes. From the univariate analysis, we first showed that vmPFC average activation during choice correlates with the usefulness of the chosen item for the congruent goal only. With the RSA, we found that the vmPFC represents items in an abstract space where distances between elements were proportional to the difference in their usefulness but only relative to the participants’ goal. Critically, such space would enable comparison across behavioral options, as other studies have evidenced in the context of food and nonfood consumables (*1*, *28*, *29*). However, humans can not only compare items according to how well they fulfill a specific goal but can also express preferences between items serving different purposes. Going back to our initial example, when asked to choose between a pair of trekking shoes and a pair of headphones, humans would answer after evaluating the shoes for how comfortable they are and the headphones for the sound quality. To carry out these decisions, the brain must maintain a metric in which options are made commensurable even if they fulfill different goals. With the cross-classification analysis, we found evidence that the vmPFC (and possibly the OFC), unlike other prefrontal regions, can support this function. This means that the vmPFC represents goal-dependent usefulness with a goal-independent currency. The computation of usefulness is goal-dependent: A wooden chair is great for burning and useless for anchoring, but the way such usefulness is represented is instead conserved across goals. For instance, an item with low usefulness for burning and another item with low usefulness for anchoring would be represented similarly, even though their uselessness is computed with respect to two different goals. This result is in accordance with a previous work by McNamee and colleagues (*28*) and suggests that the vmPFC fulfills the unique role of organizing categories of items under different goals using a common code. This analysis was limited to the prefrontal regions that showed evidence of a goal-dependent usefulness code from the searchlight. These results might therefore not be unique to the vmPFC and extend to other areas that were not considered here. A previous study using univariate fMRI had suggested a distributed representation for general hedonic rewards, which include the anterior cingulate cortex, ventral striatum, and anterior insula (*40*). Unlike this study, however, we suggest here that the vmPFC encodes value or usefulness that is computed in the context of a specific goal and is independent from hedonic attributes like monetary value (Table 1 and fig. S2).

From the univariate analysis during choice, we detected a signal in a set of brain regions commonly associated with value-based decision-making (Fig. 2) (*29*, *34*, *35*). We did not find a similar univariate signal during imagination trials, when choice was not required (Fig. 2). Notably, a handful of studies have shown value univariate signals in the absence of choice (*4*, *30*, *45*). In many of these studies [e.g., (*4*)], participants, while not performing an overt choice or valuation, were still required to think about how much it was worth to them in monetary terms. In other studies, perceptual and value estimations were interleaved in the same task [e.g., (*44*)]. Here, instead, we asked the participants to imagine how the item could be used toward a goal, without being explicitly asked to think about a scalar value. In line with this, asking participants to engage in a distracting task while viewing items and not instructing them to construct a value estimate produced a substantial weaker response in the value regions compared to when a value-based decision is required (*30*). Together, these results suggest that for a univariate signal to arise in the brain network commonly associated to value in the absence of choice, subjects must be required to perform an implicit mental valuation.

Beyond the vmPFC, the OFC and dlPFC also exhibit a neural representation compatible with a usefulness coding. The OFC is a central element of the value circuit in the brain (*2*, *34*, *35*) and is implicated in computing action outcomes during decisions (*42*, *46*, *47*). This raises the intriguing hypothesis that the representational similarity in the OFC reflects a similarity across imagined outcomes, which could then be read out by downstream regions for evaluation or choice (e.g., vmPFC). The dlPFC, on the other hand, represents the expected rewards (*48*) and the task rules (*49*). For instance, Wallis *et al.* (*49*) found a higher incidence of rule-selective neurons in the dlPFC compared with other prefrontal areas. This is consistent with our results (Fig. 5B), which indicate that item usefulness in the dlPFC uses different codes for different goals.

Using RSA combined with an approach previously used for multicell recordings in rodents (*42*), we showed that the OFC supports an integrative code of value and confidence. However, the low spatial resolution of fMRI data limits the interpretations of these findings. While our results strengthen the emerging results on the interplay between confidence and value for behavioral control (*10*, *25*, *26*, *39*, *50*), additional work is required to provide a conclusive answer.

While goal-dependent usefulness representations must account for the current goal, an item’s sensorial representation is presumably driven by its goal-independent perceptual features. We found representations fulfilling this criterion across the ventral stream in the occipital and temporal lobe—known to encode item identity and certain stimulus categorizations (*32*). In addition to a perceptual representation, visual areas like the fusiform gyrus and the ITG also encode usefulness representations of the items (Fig. 3B). A correlation between usefulness and brain activity in the occipitotemporal cortex was also evident in the univariate analysis (Fig. 2B). This activity correlated not only with congruent usefulness but also with the goal-irrelevant incongruent usefulness and monetary value (fig. S2). Since participants had already been exposed to the items in the three contexts before the fMRI session, these results can be explained with a stimulus-driven attention capture by items that are valuable in any of the proposed contexts (*51*) and are in line with previous findings showing occipitotemporal activation stimulus-driven attention orientation (*52*) and when attention is captured by value during choice even when value is task-irrelevant (*30*). This additionally resonates with growing evidence of an engagement of sensory areas in higher-level cognition (*53*–*55*).

In conclusion, our work provides new empirical evidence for how the brain adjusts neural representations in response to changes in behavioral goals. We believe that our findings nuance the commonly held view that equates value with reward or pleasantness; instead, they emphasize a role of goal-dependent usefulness representations in guiding choice and constructing value. Unlike the gamified settings in which artificial agents are usually tested (*56*), the real world does not provide easily calculable rewards akin to points on a score sheet. Humans must instead construct value on the fly, in response to changing behavioral contexts. Mapping how the brain can achieve this computational feat can provide essential clues for understanding or perhaps even designing flexible cognitive architectures.

## METHODS

### Participants

We recruited 41 volunteers from the general population (age: means ± σ^2^ = 24.1 ± 3.2 years; 19 females). All participants were right-handed, fluent in English, and had normal or corrected-to-normal vision. We excluded two participants because of anatomical anomalies that caused artifacts in the functional brain images and four participants because of excessive head motion (more than 2 mm for more than 2% of the trials). We excluded five additional participants because their behavior on days 1 and 2 was inconsistent (fig. S1). Specifically, following a criterion set in previous studies (*9*, *26*) to make sure participants were consistent and engaged with the task, participants were excluded if the logistic regression between choice and value difference on single-subject data had a slope smaller than 0.025 (see the “Experimental paradigm” section). Therefore, a total of 30 participants were included in the final analysis. The study was conducted in accordance with the Declaration of Helsinki and was approved by the Research Ethics Committee of the University College London. Before starting the experiment, all participants gave written consent. After the experiment, they were compensated for their participation.

### Experimental paradigm

The experiment took place over 2 days. On day 1, participants were shown high-resolution photos of 120 items from everyday life (Fig. 1A) on a computer screen and were asked to indicate their familiarity with each item and an estimation of its market retail price. Next, they familiarized themselves with the cover story that contextualized the upcoming task (see the Supplementary Materials). Participants were asked to picture themselves as pilots of a cargo aircraft flying over the ocean at night. A sudden engine failure required an emergency landing on a deserted island. The task was to flee the island. We devised two possible escape plans, each requiring the achievement of a separate goal. The first goal (hereafter referred to as burning; Fig. 1B) was to start a fire to be detected by a rescue team. At this point, participants were shown again the initial set of items (which, as it turns out, was the content of the cargo) and were asked to evaluate each item’s usefulness (i.e., goal-directed value) for this first goal and the confidence in such evaluation. Usefulness and confidence were indicated independently by positioning a cursor on two continuous scales displayed simultaneously on the screen under the item picture (Fig. 1C). The range of the value slider was between “not valuable” and “very valuable” whereas for confidence was between “just guessing” and “totally sure”; no number appeared on either slider. The current goal was reminded by an icon at the top left corner of the screen (Fig. 1C). The second escape plan involved using a little boat found floating near the coast. However, taking to the high seas at night was unadvisable. Therefore, the second goal was to keep the boat anchored ashore until morning (anchoring goal; Fig. 1B). Participants then saw each item once more and estimated the item usefulness and the associated confidence toward this second goal. Participants could not choose which escape plan to follow but had to evaluate the items for both goals.

On day 2, participants underwent fMRI scanning. The experiment took place over four sessions, each associated with one of the two goals in alternate order that was counterbalanced across participants. Each session consisted of 84 trials: 60 imagination trials and 24 choice trials. During imagination trials, the image of an item (from the same set of everyday-life items experienced on day 1) was presented for 5 s, during which participants were asked to imagine using it for achieving the current goal (specified at the beginning of the session and reminded throughout by an icon in a corner of the screen; Fig. 1C). No behavioral response was required during imagination trials. Across the two sessions corresponding to the same goal, all 120 items were presented. During choice trials, participants were shown a randomly selected pair of items for 3.5 s and were tasked to indicate the item they deemed most valuable for the current goal. Choice options were selected only among the items presented during the 10 most recent imagination trials so that choice trials would incentivize participants to comply during imagination trials, since choices could be made easier by considering the possibly upcoming items in isolation during imagination trials. The choice was selected by pressing a button on a joypad, which triggered the appearance of a yellow star below the chosen item, confirming the selection (Fig. 1C). All the computer tasks were created with the Python toolbox PsychoPy [www.psychopy.org; (*57*)].

### Behavioral analysis

Behavioral data were analyzed using a logistic regression. The aim of this analysis was to estimate the impact of subjective usefulness *U* and confidence *C* reported on day 1 on choices of day 2. Specifically, we sought to estimate the degree by which choice was driven by each goal (burning and anchoring; Fig. 1B), difference in usefulness between the options (Δ*U* = *U*_Right_ − *U*_Left_; measured in the context of both goals, as well as in monetary terms), confidence difference (Δ*C* = *C*_Right_ − *C*_Left_), and the interaction Δ*U* : Δ*C*. Notably, we separately estimated the effect of usefulness and confidence reported in the context of the congruent and the incongruent goal (Fig. 1, D to F). This resulted in a total of eight predictors (goal, congruent and incongruent usefulness, congruent and incongruent confidence, congruent and incongruent interaction, and monetary value), which we collectively denote as *X* = *x*_1_, …, *x*_8_. The effect of the predictors on each participant’s choice was estimated by fitting a generalized linear mixed model to the behavioral data. Accordingly, the probability of participant *k* to choose the right option (*R*) was modeled as (Fig. 1F)

$$P^{k}(X)=\frac{1}{1+e^{-{Y(X)}^{k}}}$$

with

$$Y(X)=\beta_{0}+b_{0}^{k}+(\beta_{1}+b_{1}^{k})x_{1}+\ldots +(\beta_{8}+b_{8}^{k})x_{8}$$

where β_0_ is the group intercept, β_1_, …, β_8_ the group (fixed) effects, and $b_{1}^{k},\ldots ,b_{8}^{k}$ are the subject-specific random effects, which have prior distribution

$$b_{i}^{k}\sim N(0,\sigma_{b}^{2})$$

This is equivalent to the Wilkinson notation

$$\text{Choice}\sim 1+x_{1},\ldots ,x_{8}+(\text{subject})$$

The model was estimated with MATLAB (MathWorks, Natick, MA, USA) using the built-in function fitglme.

### fMRI data acquisition and preprocessing

Brain imaging data were acquired at the Wellcome Trust Centre for Human Neuroimaging using a Siemens (Erlangen, Germany) Prisma 3.0-T MRI scanner with a 64-channel head coil. We measured BOLD activity over four experimental sessions with T2*-weighted echoplanar images (EPI) that were acquired with a sequence optimized for brain regions near the OFC and the amygdala (voxel size = 3 mm by 3 mm by 3 mm; matrix size = 64 × 72; repetition time (TR) = 3.36 s; echo time (TE) = 30 ms; slice tilt = −30). We acquired 225 volumes in each session, resulting in a total session duration of 12.6 min. We discarded the first five volumes of each session, during which no stimuli were presented, to allow for the stabilization of the magnetic field. Forty-eight slices were acquired for each volume. Subjective T1-weighted MPRAGE anatomical scan and field maps were acquired for each subject.

Image preprocessing was carried out with the MATLAB toolbox Statistical Parametric Mapping (SPM12; www.fil.ion.ucl.ac.uk/spm) and custom code. Raw EPIs were slice time-corrected, realigned, and then unwarped using the data from field maps. No further preprocessing was needed for the multivariate analyses, which were performed in native space. For the univariate analysis, each subject’s anatomical image was further mapped onto a template of tissue probabilities with a nonlinear deformation map and then segmented into gray and white matter, cerebrospinal fluid, bone, soft tissue, and air images. The same deformation map was then used to normalize the EPIs to the MNI template, which were lastly smoothed with a Gaussian kernel with full width at half maximum (FWHM) of 8 mm.

### Univariate analysis

Univariate analyses were performed with SPM12 and custom code. In our main general linear model (GLM), the first regressor was an indicator function of the onsets of the imagination trials with three parametric modulators: the usefulness and confidence collected on day 1 under the same goal (i.e., congruent usefulness and confidence) and the estimated monetary value. The second regressor was an indicator function of the onsets of choice trials, parametrically modulated by the difference between the congruent usefulness estimations of the chosen and unchosen item. We created an additional GLM identical to the one above but using the usefulness and confidence estimates collected on day 1 under a different goal (i.e., incongruent usefulness and confidence). We have also constructed a third GLM that includes all the regressors from the two GLMs described above (table S3). Regressors were not orthogonalized but were left to compete for variance. In both GLMs, we also corrected for motion artifacts by including six subject-specific parameters from image realignment (corresponding to three rigid-body translations and three rotations) as covariates of no interest. All regressors were then convolved with a canonical hemodynamic response function. A high-pass filter with a cutoff of 1/128 Hz was applied to the time series to remove slow drifts. All presented clusters were detected with a cluster-defining threshold of *P* < 0.001 and FWE-corrected for multiple comparisons at *P* < 0.05.

### Multivariate analysis

#### Representational similarity analysis

RSA is a multivariate analysis technique suited to investigate the neural representation of brain regions. In this framework, hypotheses about the neural representation are specified in terms of RDMs, which encode the pairwise dissimilarity (or distance) between experimental conditions predicted by a model. For instance, if we were testing for brain regions encoding the color of a stimulus, the model RDM would prescribe low distance between stimuli with similar color and high distance between stimuli with very different colors. Hypotheses are then tested by comparing model RDMs to brain-based RDMs that encode the pairwise distance, computed as 1 − Pearson’s *r* (*21*, *22*), between brain activity patterns elicited in a given ROI by the different experimental stimuli. This second-level similarity, which was computed in terms of Spearman’s rank correlation coefficient ρ, allowed inferring to what extent the activation pattern in a specified ROI correlated with a model prediction. In the present study, the RSA was carried out with a dedicated MATLAB toolbox (*21*) and custom code. Our task involved evaluating 120 different items twice, once for each goal, resulting in 240 conditions for each of which we computed a separate β-map with a dedicated GLM on unsmoothed data in the subjective native space. Before all analyses, β-maps were spatially prewhitened within the considered ROI to maximize the reliability of our inferences (*58*). Since the RDMs encoded all pairwise distances in a set of 240 objects, they had dimensions of 240 × 240.

For the representation of item identity, the model RDM prescribed low distance between presentations of the same item even under different goals and high distance between distinct items. The distance between items *i* and *j* was thus formalized as *d_ij_* = 1 − δ*_ij_*, where δ is the Kronecker delta; the distance is thus 0 if the indices *i* and *j* coincide and 1 otherwise. To test for representations based on subjective valuations, we defined a model in which the distance was the absolute value difference between pairs of stimuli (i.e., *d_ij_* = ∣*DV*∣*_ij_* = ∣ *V_i_* − *V_j_*∣). A similar representation based on subjective confidence instead of valuation (i.e., *d_ij_* = ∣*DC*∣*_ij_* = ∣ *C_i_* − *C_j_*∣) was also considered. To create the model RDM of material composition, we asked a different set of participants to estimate, for each item, the compositional percentage of metal, wood, plastic, and fabric. Compositional distance was then defined as the average Euclidean distance in this four-dimensional space. Notably, similarly to the representation of item identity, this prescribed high similarity between subsequent presentations of the same item. Hence, these representations were partially correlated. To rule out that the extent of material composition representation reported by the searchlight was inflated by the representation of item identity, we excluded the matrix elements corresponding to item self-similarity across goals, therefore making the two representations approximately orthogonal. Once the model representations were created, we tested them following different pipelines for ROI-based and searchlight approaches.

#### Searchlight

The searchlight analysis was performed within subjective gray matter masks. In line with previous approaches (*59*), these masks were defined as the set of voxels with probability of including gray matter exceeding 0.3 according to the tissue segmentation step. A spherical ROI with a radius of 9 mm (3 voxels) was defined around each voxel in the gray matter mask. One brain-based RDM was built in each of these spherical ROIs. Model and brain-based RDMs were then reshaped to a vector and compared, yielding a correlation coefficient (Spearman’s ρ) that was assigned to the center of the sphere. Therefore, this procedure ultimately created a subjective correlation map, which encoded the correlation between model and brain representation across the whole gray matter mask. The maps were lastly normalized to MNI space with the deformation map from the tissue segmentation and then smoothed with a Gaussian kernel with FWHM of 8 mm. Group-level statistics was performed with nonparametric cluster-level permutation test implemented in SnPM (statistical nonparametric mapping) (*31*), with a cluster detection threshold at *P* < 0.001 and no variance smoothing. Using a standard procedure, clusters were considered significant if they survived a FWE correction at *P* < 0.05 (*60*).

#### ROI analysis

For the ROI-based analysis of confidence, prefrontal ROIs were defined within spherical masks with a radius of 10 mm around the peak activation of the prefrontal clusters identified by the searchlight (from the usefulness searchlight, vmPFC: [−10, 54, 2], OFC: [−32, 38, −14], and dlPFC: [−40, 50, 12]), which were created with the toolbox MarsBar. Crucially, we did not conduct multivariate testing for usefulness within these ROIs, as it would imply circularity in the inference. Instead, we tested for confidence, which is nearly orthogonal to subjective value (Table 1). To determine whether an ROI presented a model-led representation, brain- and model-based RDMs were reshaped to a vector and then compared in terms of the Spearman rank correlation. Correlation coefficients ρ were then tested for significance at the group level with the Wilcoxon signed-rank test, a nonparametric statistical test that relaxes the normality assumption often violated by correlation coefficients.

To gather indications on whether representations of usefulness and confidence were supported by the same neural circuitry, we capitalized on an approach previously used in the study of cell ensembles in rodents (Fig. 4C) (*42*). Specifically, from the multivoxel activity in the ROI, we computed two “participation” vectors (*42*) containing a measure of the strength with which each voxel encoded either usefulness or confidence. Each entry of the vectors was the unsigned coefficient of a linear regression of the activity in a voxel against the usefulness or the confidence of the presented item. A positive correlation (Spearman’s ρ) between the two vectors would suggest that voxels participate similarly in encoding value and confidence, which is indicative of a shared representation rather than overlapping but distinct representations (*42*). Since we had no hypotheses about the distribution of these correlation coefficients, statistical inference was carried out with a nonparametric permutation test in which we repeated the same analysis 10,000 after permuting the trial labels. The probability that the observed correlations were extracted from the null distribution was computed as the relative number of permutations that presented a Wilcoxon’s *z* statistic value higher than the one obtained from the original (unpermuted) analysis.

#### Neural code generalization across goals

We sought to investigate to what extent goal manipulations affected the neural code of usefulness. To do so, we sought to assess whether a pattern classifier trained to predict usefulness from brain activity in the context of one goal could generalize to predict value for the alternative goal. Since there was a significant difference between the usefulness reported by subjects under different goals (higher usefulness for burning than for anchoring, *P* < 0.001), there was a potential goal bias on the models. To overcome this bias, following previously used methods (*28*), the data were split into high and low usefulness at a subject level, thus eliminating the correlation between goal and usefulness. The schematics of this procedure are detailed in Fig. 5A. Specifically, we built two support vector machines (SVMs; one for each goal) for each subject and ROI to classify the multivoxel ROI activity into low or high value. The frequent inconsistencies between subjective valuations (acquired on day 1) and choice preferences (acquired on day 2; fig. S1) suggest that the subjective internal estimation of value might be prone to volatility across the two experimental sessions. To minimize the detrimental effect of these inconsistencies on the classifiers’ accuracy, we thus retained only the items in the highest and lowest value quartile. To train the SVM, we used the function fitcsvm implemented in MATLAB. To assess whether the classifiers generalized to the alternative goal, we compared the accuracy they achieved on data from the same goal as the training set, computed with a 10-fold cross-validation procedure, with that achieved in classifying data acquired under the alternative goal context. Statistical testing on single-subject classifier accuracies was performed with one-sided Wilcoxon signed-srank tests.

## SUPPLEMENTARY MATERIALS

Supplementary material for this article is available at http://advances.sciencemag.org/cgi/content/full/7/15/eabd5363/DC1

## Appendix

View/request a protocol for this paper from *Bio-protocol*.
